# Virginia M. Bowden (1940-2022)

**DOI:** 10.5195/jmla.2022.1579

**Published:** 2022-07-01

**Authors:** Janna C. Lawrence

**Affiliations:** 1 janna-lawrence@uiowa.edu, Director, Hardin Library for the Health Sciences, University of Iowa, 600 Newton Road, Iowa City, Iowa 52242.

Virginia M. Bowden, MSLS, PhD, AHIP, FMLA, director emeritus of the Briscoe Library at the University of Texas Health Science Center at San Antonio (now UT Health San Antonio) passed away on May 2, 2022, at the age of eighty-two. She was at her home in San Antonio surrounded by her daughters, Sharon and Ellen, and her beloved cat Al. Her husband of sixty-one years, Dr. Charles Bowden, passed away just two months before her on March 1, 2022.

Virginia was valedictorian of her class at Stephen F. Austin High School in Houston, and at the University of Texas at Austin, she majored in math and education and took some of the first classes in computer programming. After graduating Phi Beta Kappa in just three years, Virginia worked as a computer programmer for organizations as diverse as Texaco and Bamberger's Department Store. In 1960 she married Charles Bowden, who went to medical school at Baylor University College of Medicine in Houston and did his internship and residency in Chicago and New York City. Virginia earned her master's degree in library science in 1970 at the University of Kentucky in Lexington, Kentucky, while Charles served in the US Public Health Service there.

After later settling in San Antonio, Virginia accepted a position at the very new University of Texas Health Science Center at San Antonio (UTHSCSA) as a library systems analyst. She was soon appointed assistant to the director and then associate library director before being selected as library director in 1985, a position she held until her retirement in 2003.

Virginia earned a doctor of philosophy degree in library and information science from the University of Texas at Austin in 1994. Her dissertation, entitled “Current Monograph Collections: Patterns of Ownership and Use in Four Academic Health Sciences Libraries,” combined her interests in collection development and computers and made use of collection and circulation data from four similar health sciences libraries.

Her passion for computers and library automation was apparent throughout her career. In 1971, she managed the library's adoption of PHILSOM (Periodical Holdings in the Libraries of Schools of Medicine), a computer-based serials control system developed by the Washington University School Medicine Library. In 1978, she directed the implementation of the Computer Library Systems, Inc. (CLSI) circulation system, shared with another San Antonio institution, Trinity University. Under her direction, the UTHSCSA Library in 1983 became the second library, after Georgetown University, to implement Georgetown's Library Information System (LIS), an early integrated library system designed for health sciences libraries. The switch to an online catalog, locally called the Biomedical Library Information System (BLIS), coincided with the move to a new, larger library building, and when that move was made, the card catalog was left behind [1].

In 1990, the Medical Library Association (MLA) awarded Virginia the third Louise Darling Medal for Distinguished Achievement in Collection Development in the Health Sciences, recognizing “her creative approaches, publications, and innovating use of machine-readable files for collection decision making and cooperative collection development” [2]. Virginia was a nominee for MLA president-elect in 1994 and held numerous appointments in MLA and in the South Central Chapter/MLA. She was granted MLA fellowship status in 2000.

Virginia's mentorship and love of libraries extended beyond health sci-ences including service as president of the Friends of the San Antonio Public Library from 1989-90. In 2003, she was awarded the Julia Grothaus Award by the Bexar Library Association for her dedication to library service in Bexar County, where San Antonio is located. Virginia also served on the Advisory Council of the University of Texas School of Information.

In 1992, Virginia and Charles established the Bowden Massey Foundation, which has funded several library-related projects, including the Virginia and Charles Bowden Professor of Librarianship at the University of Texas at Austin, the Bowden Conference Room at the Briscoe Library, and MLA's David A. Kronick Traveling Fellowship. Later in her career, Virginia focused on access to health information, particularly in the Lower Rio Grande Valley at the southernmost tip of Texas. This included surveying physicians on their use of MEDLINE and other databases; introducing health care professionals to Grateful Med, the floppy disk-based predecessor to PubMed; and promoting MedlinePlus to health care professionals and consumers. Under Virginia's leadership, the UTHSCSA Library developed a robust circuit librarian program in South Texas. Two of the circuit librarians were recognized with the Michael E. DeBakey Library Services Outreach Award for serving rural or underserved populations: Mary Jo Dwyer in 1993 (the first year it was awarded) and Greysi Reyna in 2009.

Virginia was especially proud of her six grandchildren and always looked forward to family gatherings. She and Charles enjoyed skiing, spending time in the Colorado mountains where they owned a vacation home, and traveling together to over fifty countries on six continents.
